# The Royal College of Surgeons

**Published:** 1898-11-26

**Authors:** 


### THE ROYAL COLLEGE OF SURGEONS.

The annual meeting of the Fellows and Members of the Royal College of Surgeons passed off without harm to any one, and we might truthfully say without much benefit either. A resolution affirming a principle which had already been affirmed year after year—namely, that the members of the college should have some voice in the management of its affairs—was affirmed again once more, which, although no doubt satisfactory to the mover and seconder of the resolution, does not mean much. There does seem some hope, however, that the Members will before long receive permission to wear an academic gown! Mr. Brindley James and Dr. F. H. Alderson are the gentlemen who are responsible for the resolution in favour of the "privilege" of wearing this garment being granted to Members, and the Council seems not unwilling to take steps to meet this demand. This is at least a harmless placebo!

